# Phylosymbiosis and functional redundancy in the *Drosophila* (Diptera: Drosophilidae) gut microbiome and its implications for host fitness

**DOI:** 10.1093/jisesa/ieaf114

**Published:** 2026-01-27

**Authors:** Jahir Muñoz-Hernández, Ignacio Peralta-Maraver, Grisel Cavieres, Ignacio Gutiérrez-Cortés, Enrico L Rezende, Daniela S Rivera

**Affiliations:** GEMA Center for Genomics, Ecology and Environment, Facultad de Ciencias, Ingeniería y Tecnología, Universidad Mayor, Santiago, Chile; Programa de Doctorado en Ecología Integrativa, Universidad Mayor, Santiago, Chile; Center of Applied Ecology and Sustainability (CAPES), Santiago, Chile; Departamento de Ecología, Universidad de Granada, Granada, Spain; Research Unit Modeling Nature (MNat), Universidad de Granada, Granada, Spain; Departamento de Zoología, Facultad de Ciencias Naturales y Oceanográficas, Universidad de Concepción, Concepción, Chile; Center of Applied Ecology and Sustainability (CAPES), Santiago, Chile; Extreme Ecosystem Microbiomics & Ecogenomics Lab, Facultad de Ciencias Biológicas, Pontificia Universidad Católica de Chile, Santiago, Chile; Center of Applied Ecology and Sustainability (CAPES), Santiago, Chile; Facultad de Ciencias Biológicas, Pontificia Universidad Católica de Chile, Santiago, Chile; GEMA Center for Genomics, Ecology and Environment, Facultad de Ciencias, Ingeniería y Tecnología, Universidad Mayor, Santiago, Chile

**Keywords:** gut microorganism, host reproduction, microbial function, holobiont, symbiosis

## Abstract

The gut microbiome plays a fundamental role in host ecophysiology. Numerous studies have examined microbiome composition and functionality to understand the ecological and evolutionary factors shaping host–microbe interactions. However, the consequences of these patterns for animal ecology remain poorly understood. Here, we examined how variations in the gut microbiome influence fitness differences among *Drosophila* species sharing a common dietary niche. Using 16S rRNA gene sequencing, we analyzed the gut microbial taxonomy and predicted functional profiles of 4 *Drosophila* species collected in central Chile. Our results revealed a strong signal of phylosymbiosis in the microbial taxonomy, while functionality was highly redundant across the studied fly species. Functional biomarkers analysis indicated that the gut microbiome supports the nutritional requirements of *D. simulans* (Sturtevant), *D. hydei* (Sturtevant), and *D. repleta* (Wollaston); whereas, this was less evident in *D. melanogaster* (Meigen). To assess the potential contribution of the microbiome to host performance, we compared egg-to-adult viability between 2 species with the greatest physiological divergence: *D. simulans* and *D. hydei*. Notably, *D. simulans* exhibited significantly higher egg viability and shorter development time than *D. hydei*. Strikingly, the *D. simulans* microbiome contained more taxonomic and functional biomarkers previously demonstrated to enhance fly fitness, whereas the *D. hydei* microbiome harbored taxa and functions potentially detrimental to host performance. These findings suggest that the gut microbiome contributes to host fitness and may shape the evolutionary ecology of *Drosophila* species, with broader implications for community dynamics, including interspecific competition and species displacement.

## Introduction

The gut microbiome plays a crucial role in the overall health of organisms, influencing a wide range of physiological processes, from digestion and immunity to metabolism and behavior (Wong et al. 2017, Fontaine et al. 2022). Moreover, tolerance to environmental stressors (eg thermal variability and food availability) and animal fitness (eg fecundity, lifespan, and developmental times) are also influenced by the gut-associated microbiome (Keebaugh et al. 2019, Li et al. 2019, Walters et al. 2020, Shu et al. 2021). These effects arise because the microbiome produces various metabolites that benefit its hosts at multiple biological levels. Some examples include supplying amino acids to the host (Kim et al. 2021), providing vitamins necessary for mitochondrial co-enzymes (Gnainsky et al. 2021), or generating fatty acids that promote gene expression that regulate different cellular processes (eg mitochondrial activity) (Clark and Mach 2017).

In recent years, the study of microbiome variability across species has gained significant attention, as it provides insights into the ecological and evolutionary factors shaping host-microbe interactions. In this way, phylogenetic relatedness is expected to correlate with the taxonomic composition of gut microbiomes among related host species (phylosymbiotic relationship) (Brooks et al. 2016). However, while some studies have suggested that more phylogenetically related host species tend to exhibit greater similarities in microbial taxonomic composition (Brooks et al. 2016, Ravigné et al. 2022), others have found no such correlation (Adair et al. 2020, DuBose et al. 2025), indicating that gut microbiome phylosymbiosis is not universally applicable across animal clades (Lim and Bordenstein 2020, Mallott and Amato 2021). Considering that the phylogenetically related host species should exhibit greater similarity in their trophic niches (ie phylogenetic niche conservatism hypothesis) (Losos 2008), then closely related hosts may depend on symbionts performing comparable metabolic functions, even if their microbiomes differ taxonomically. This phenomenon appears to be widespread across microbial communities, even at local scales, genetically distinct microorganisms tend to coexist in consortia that fulfill similar functional roles (Louca, Jacques, et al. 2016, Louca, Parfrey, et al. 2016, Nelson et al. 2016, Vanwonterghem et al. 2016, Louca et al. 2018, Tully et al. 2018). Such functional redundancy has been proposed to explain the stability and resilience of the human gut microbiota, which exhibits considerable taxonomic variability even across individual hosts (Tian et al. 2020). However, the extent to which this phenomenon occurs in other animal species remains largely unexplored, although some studies have addressed it (Bost et al. 2018, Estrada-Peña et al. 2020).

Moreover, because variations in both the composition and function of gut microbiomes are associated with specific host fitness traits (Matthews et al. 2021), it is expected that microbial traits will be associated with fitness differences across host species. This is particularly relevant because it may explain how microbiomes influence the evolutionary success of host species in competitive interactions. For instance, host species with specific microbial biomarkers may exhibit enhanced adaptability and competitive advantage, enabling them to outcompete and potentially displace related species (Zhang et al. 2022, Wei et al. 2024). Most research linking specific bacterial taxa in the microbiome to host fitness has focused on vertebrates (Rosshart et al. 2017, Meijers et al. 2019, Davidson et al. 2021). In contrast, relatively few studies have explored the relationship between specific microbiome symbionts and fitness traits in other organisms, such as insects (Pais et al. 2018, Matthews et al. 2021). To date, only a few studies have investigated the association between specifics microbiome functional traits and fitness differences across insects’ host species (Zhang et al. 2022). Consequently, the role of the microbiome in determining competitive success among host species remains unclear.

This study examines the taxonomic and functional variation in the gut microbiomes of four *Drosophila* species that coexist geographically and occupy similar ecological niches in terms of food source and breeding sites: *D. simulans* (Sturtevant), *D. melanogaster* (Meigen), *D. hydei* (Sturtevant), and *D. repleta* (Wollaston) (Diptera: Drosophilidae). We assessed how the microbiome’s alpha and beta diversity varied across the species in the wild, identifying species-specific bacterial taxa and functional traits. Subsequently, we cultured the two phylogenetically most distant fly species, which exhibit the greatest reported physiological differences (eg fecundity and development time) (Atkinson 1979, Markow and O’Grady 2005), in the laboratory to assess the relationship between taxonomic and functional diversity of microbiomes with host egg-to-adult viability and development time. Our work advances the understanding of how microbiomes support host fitness, facilitate adaptation to environmental changes, and support key ecological and evolutionary processes.

## Materials and Methods

### Fly Capture and Maintenance

Adult flies of *D. simulans*, *D. melanogaster*, *D. hydei*, and *D. repleta* were captured in central Chile (33°26′S; 70°39′W at 500 m above sea level) between December 2023 and April 2024 using banana and yeast traps. Captured individuals were brought to the laboratory for taxonomic identification based on morphological characters (Werner et al. 2020). We conducted microbiome analyses on adults of each collected species. We focused on the microbiome of female flies because of their ecological importance in population dynamics and their direct link to offspring viability (Li et al. 2022, Kappeler et al. 2023). For this reason, females were selected for *D. hydei* and *D. repleta*. In contrast, males were used for *D. simulans* and *D. melanogaster* due to the morphological indistinguishability of females in these species. Importantly, previous studies have reported no significant sex-based differences in microbiome composition across several *Drosophila* species, including *D. simulans*, *D. melanogaster*, and *D. hydei* (Wong et al. 2013, Adair et al. 2020), and similar patterns have been suggested for *D. repleta* (Comeault et al. 2025). On this basis, we considered the microbiome profiles of wild males to be representative of those of females in *D. simulans* and *D. melanogaster*, thereby ensuring comparability with the female-derived profiles of *D. hydei* and *D. repleta*.

We measured fitness traits in *D. simulans* and *D. hydei*, 2 phylogenetically distant fly species that differ in their microbial composition (Adair et al. 2020) and life-history traits (Atkinson 1979, Markow and O’Grady 2005). Therefore, populations of these species were established in the laboratory. Two groups of each species were set up, each with approximately 50 individuals. Groups were reared in controlled conditions at a constant ambient temperature of 21 °C and a photoperiod of 16:8 h L:D, housed in Plexiglas cages (27 × 21 × 16 cm^3^) containing plates with Burdick culture medium (Burdick 1955). To maintain a discrete-generation regime, flies were allowed to oviposit for 36 h on fresh food plates placed in each cage. The collected eggs were then transferred to 250 ml bottles with culture medium (approx. 100 eggs per bottle, to prevent overcrowding effects). Emerging adults were transferred to a new cage. Population size within each cage varied between roughly 1,000 and 1,500 breeding adult individuals, and we maintained in three replicate cages that were pooled every generation to minimize the loss of genetic variation due to drift.

### Microbiome Analysis

#### DNA Extraction and 16S rRNA Gene Sequencing

Flies were surface sterilized by vortexing them in 0.5% sodium hypochlorite (in 1× PBS) for 1 min, followed by 3 washed in 1× PBS. Then, the flies were stored in RNAlater until further use. Intestinal dissections (from crop to hindgut) were performed as described by Tauc et al. (2014), pooling 10 intestines per replicate (5 replicates per treatment). DNA was immediately isolated using the DNeasy Blood and Tissue Kit (Qiagen). To ensure DNA was free of RNA contamination, 10 µl of RNase A (10 mg/ml) was added per sample. DNA quality was assessed via 1% agarose gel electrophoresis. Subsequently, DNA samples were sent to the Zymo Research Central Laboratory (Irvine, California, United States) for sequencing.

#### Targeted Library Preparation

The DNA samples were prepared for targeted sequencing with the Quick-16STM Plus NGS Library Prep Kit (Zymo Research, Irvine, California, United States). These primers were custom designed by Zymo Research to provide the best coverage of the 16S gene while maintaining high sensitivity. The primer set used in this project was Quick-16STM Primer Set V3-V4 (Zymo Research, Irvine, California, United States).

The sequencing library was prepared using an innovative library preparation process in which PCR reactions were performed in real-time PCR machines to control cycles and, therefore, limit PCR chimera formation. The final PCR products were quantified with qPCR fluorescence readings and pooled together based on equal molarity. The final pooled library was cleaned up with the Select-a-Size DNA Clean & ConcentratorTM (Zymo Research, Irvine, California, United States), then quantified with TapeStation (Agilent Technologies, Santa Clara, California, United States) and Invitrogen Qubit 1× DSDNA High-Sensitivty Assay Kits (Thermo Fisher Scientific, Waltham, Washington, United States).

#### Control Samples

The ZymoBIOMICS Microbial Community Standard (Zymo Research, Irvine, California, United States) was used as a positive control for each DNA extraction, if performed. The ZymoBIOMICS Microbial Community DNA Standard (Zymo Research, Irvine, California, United States) was used as a positive control for each targeted library preparation. Negative controls (ie blank extraction control, blank library preparation control) were included to assess the level of bioburden carried by the wet-lab process.

#### Sequencing and Bioinformatics Analysis

The final library was sequenced on Illumina NextSeq 2000TM with a p1 (cat 20075294) reagent kit (600 cycles). The sequencing was performed with 30% PhiX spike-in. Unique amplicon sequences were inferred from raw reads using the Dada2 pipeline (Callahan et al. 2016). Chimeric sequences were also removed with the Dada2 pipeline. Taxonomy assignment was performed using Uclust from Qiime v.1.9.1. Taxonomy was assigned with the Zymo Research Database, a 16S database that is internally designed and curated, as a reference.

#### Fitness in *D. simulans* and *D. hydei*

We estimated fitness by quantifying the egg-to-adult viability in *D. simulans* and *D. hydei*. For *D. simulans*, we used flies from the fifth laboratory generation (F5) to ensure species identity and prevent contamination with *D. melanogaster* (see above). Although such a period was unnecessary to identify *D. hydei*, we assessed its fitness in the F5 laboratory generation to match the acclimation time of *D. simulans*.

*D. simulans* reaches sexual maturity at two days posteclosion, while *D. hydei* takes 9 d at 24 °C (Pitnick et al. 1995, Markow and O’Grady 2005). Since the acclimation temperature was 21 °C, we collected eggs from 3- to 13-d-old *D. simulans* and 10- to 20-d-old *D. hydei* flies. To quantify egg-to-adult viability, clean food plates were placed in maintenance cages, allowing flies to oviposit for 24 h. Then, eggs were collected using a microscope, Leica S6. From each plate, 20 eggs were transferred to vials with fresh medium. Fifteen vials per species (∼600 eggs in 30 vials) were placed in a climatic chamber under identical conditions: 21 °C, 16:8 h L:D photoperiod. Emergence of adults was monitored every 24 h (between 9:00 AM and 11 AM) until the last eggs emerged or alternatively after 50-d. Following Alruiz et al. (2023, 2024), we defined egg-to-adult viability as the number of surviving individuals divided by their average developmental time, yielding a rate of adult emergence per unit of time (ie eggs hatching per day). This metric is consistent with the developmental time index proposed by Vacek (1982). Development time was estimated as the number of days each individual required to hatch. For each vial, we calculated a weighted mean developmental time by multiplying the number of individuals hatched on each day by the corresponding day, summing these values, and dividing by the total number of individuals. Each vial was treated as a single replicate (*n* = 15).

### Statistical Analysis

Microbial diversity analyses were performed using the R package Microeco version 1.9.0 (Liu et al. 2021). Taxonomic diversity across *Drosophila* species was estimated using the Chao1, Shannon and Simpson indices using the R package “vegan” (version 2.4-4.) (Oksanen et al. 2017). To predict the functional capacity of microbial communities, we used the Tax4Fun2 approach (Wemheuer et al. 2020) implemented within the R package Microeco (Liu et al. 2021), based on the Ref99NR database. Microbial functional diversity was assessed using the Shannon and Simpson indices. Although both indices account for richness and evenness, the Shannon index is more sensitive to rare taxa, whereas the Simpson index emphasizes dominant taxa (Xia et al. 2018); thus, providing a comprehensive view of alpha diversity for comparing microbial communities. To assess the normality assumption, we applied the Shapiro–Wilk test. When normality was satisfied, statistical differences were evaluated using ANOVA; otherwise, the Kruskal–Wallis test was employed. Importantly, the symbionts *Wolbachia* sp. and *Spiroplasma* sp. are intracellular bacteria that inhabit organs other than the gut (eg reproductive tissues) (Werren et al. 1995, Xie et al. 2014). Therefore, these bacteria were excluded from all analysis, consistent with previous studies (Ye et al. 2017, Adair et al. 2020).

To compare differences in the microbial composition and functional traits across of host species, we employed non-metric multidimensional scaling (NMDS) ordination based on the Bray-Curtis index, implemented through the vegan package. Since the NMDS ordination depends on the initial configuration of communities in multidimensional space, we iteratively fitted the model through 1,000 permutations to identify the optimal ordination with the best goodness of fit (Oksanen et al. 2017). Additionally, we determined the centroid location of the gut microbiomes and calculated the 95% confidence interval (CI) around the centroids in the multidimensional space. The 95% CI was calculated using the *ordihull* function in the vegan package (Oksanen et al. 2017).

Differences in community composition and functional traits between the microbiomes of the *Drosophila* species were tested using permutational multivariate analysis of variance (PERMANOVA) (Anderson 2001) using Bray–Curtis distance matrix. Additionally, a linear discriminant analysis (LDA) based on effect size (LEfSe; Segata et al. 2011) was performed to identify microbiome functional traits with differential abundance between the four fly species, using the Kruskal–Wallis test and an LDA score >3. For *D. simulans* and *D. hydei*, differences in egg-to-adult viability rates and development times were assessed using the Wilcoxon test. Then, we performed LEfSe analysis to level taxonomic (ASVs) and functional between *D. simulans* and *D. hydei* to identify microbial biomarkers, which were used to predict how the gut microbiome influences host fitness traits. All analyses were performed in R version 4.3.2 (R Development Core Team, 2020, Vienna, Austria).

## Results

### Microbiome Taxonomic Composition Across *Drosophila* Species

We obtained 37,693,406 reads from 16S rRNA gene sequencing protocols (averaging approximately 1,256,447 reads per sample across 30 samples and six treatments), resulting in 273 bacterial ASVs. Rarefaction curves indicated that most of the ASVs were detected in the samples (Supplementary Fig. S1). In addition, all the samples showed high values of Good’s coverage index (≥98.98%), reflecting that the sequencing depth was adequate (Supplementary Table S1).

**Fig. 1.**
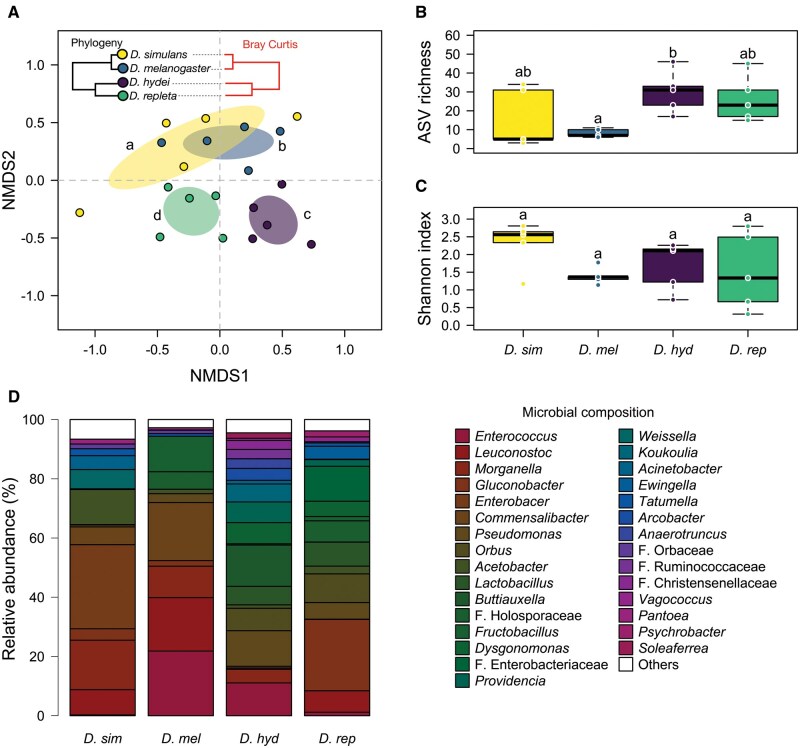
Gut microbial diversity and composition among 4 wild *Drosophila* species. A) Non-metric multidimensional scaling (NMDS) based on a Bray-Curtis distance matrix. Different letters indicate significant differences between species, as determined by PERMANOVA with pairwise comparisons. The figure shows the correspondence between host phylogeny and the microbiome similarity cluster across species, measured using the Bray–Curtis similarity index. B) Microbial richness and C) Shannon diversity index of gut microbial communities of flies. Different letters represent significant differences among flies, determined using the one-way ANOVA test. D) Relative abundance of bacterial genera in each *Drosophila* species.

The NMDS ordination model based on the ASVs composition across sampled microbiomes showed a high goodness of fit between the distances in the ordination against the original data (stress value = 0.19, linear fit *R*  ^2^ = 0.80, nonmetric fit *R*  ^2^ = 0.96). The analysis of beta diversity revealed significant vari­ation in microbial community composition among host species (PERMANOVA: 𝐹 = 1.95, 𝑅^2^ = 0.26, 𝑃 < 0.01;  Fig. 1A). The NMDS analysis showed a clear clustering of microbiome composition that reflected host phylogeny (Fig. 1A): *D. simulans* and *D. melanogaster* clustered closely together, whereas the gut microbiomes of *D. hydei* and *D. repleta* exhibited greater similarity to each other. Pairwise PERMANOVA analyses further confirmed that *D. hydei* and *D. repleta* shared more similarities in their microbial communities (PERMANOVA: 𝐹 = 1.56, 𝑅^2^ = 0.16, 𝑃 = 0.04; Fig. 1A), while *D. melanogaster* and *D. repleta* showed the lowest microbial similarity (PERMANOVA: *F* = 2.31, 𝑅^2^ = 0.22, 𝑃 = 0.01). The alpha diversity analysis revealed significant differences in the species richness [One-way ANOVA: 𝐹(3,16) = 3.84, 𝑃 = 0.03; Fig. 1B], with *D. hydei* exhibiting higher richness than *D. melanogaster*. However, no significant differences were observed in bacterial diversity among wild flies [One-way ANOVA: Shannon index: 𝐹(3,16) = 1.51, 𝑃 = 0.24; Simpson index: 𝐹(3,16) = 0.77, 𝑃 = 0.52] (Fig. 1C). The relative abundance analysis identified 6 bacterial phyla, with Proteobacteria being the most abundant across all fly species. At the genus level, *Enterobacter* was the most prevalent in *D. simulans* (28.39%) but was scarcely detected in *D. repleta* (0.07%) and *D. hydei* (0.02%), and absent in *D. melanogaster*. In contrast, *Enterococcus* was most abundant in *D. melanogaster* (21.82%), *Buttiauxella* in *D. hydei* (13.92%), and *Gluconobacter* in *D. repleta* (24.09%) (Fig. 1D). Relative abundance at the individual sample level showed high intraspecific variability (Supplementary Fig. S2). Nevertheless, the same overall pattern emerged: with *D. simulans* and *D. melanogaster* showing greater compositional similarity to each other, while *D. hydei* and *D. repleta* also display higher similarity between them (Supplementary Fig. S2).

### Microbiome Functional Traits Across *Drosophila* Species

**Fig. 2.**
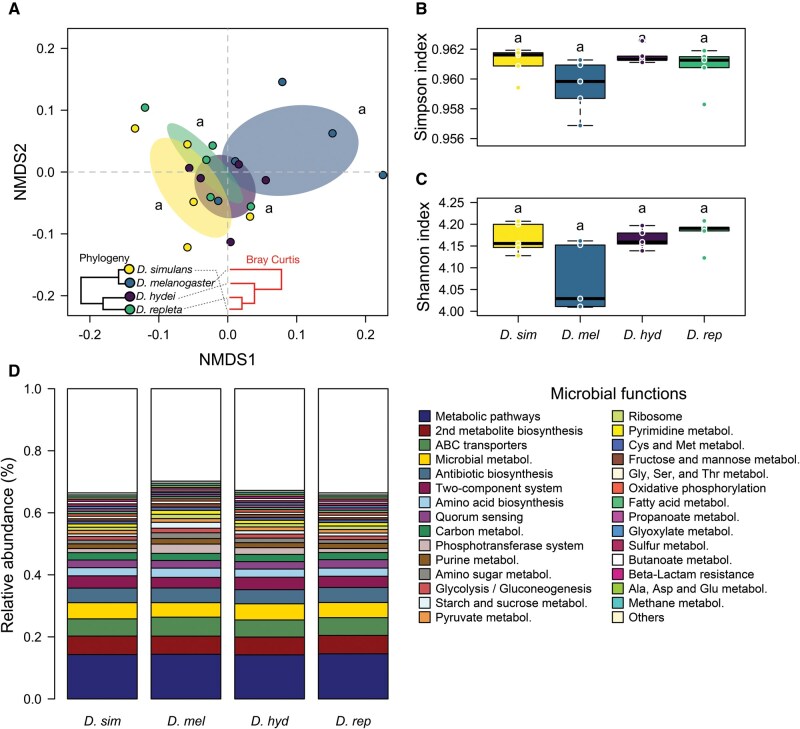
Diversity and composition of microbial functions among four wild *Drosophila* species. A) Non-metric multidimensional scaling based on a Bray–Curtis distance matrix. The figure shows the correspondence between host phylogeny and the microbiome functional similarity cluster across species, measured using the Bray–Curtis similarity index. Different letters indicate a significant difference between fly species, as determined from PERMANOVA with pairwise comparisons. B) Simpson and C) Shannon diversity indices of microbial functions in flies. Different letters represent a significant difference among flies, determined using Kruskal–Wallis test. D) Relative abundance of the bacterial functions at level 3 in each *Drosophila* species.

The NMDS ordination based on microbiome functional traits across *Drosophila* species showed a high goodness of fit between ordination distances and the original data (stress value = 0.07, linear fit *R*^2^ = 0.99, nonmetric fit *R*^2^ = 0.97). The ordination revealed markedly lower dispersion for functional traits compared to taxonomic composition (see axis ranges in Fig. 1A vs. Fig. 2A), and no significant differences in microbiome functional composition were detected among host species (pairwise PERMANOVA adjusted *P *> 0.1). Moreover, clustering of microbiome functional similarity did not correspond to host phylogeny. The alpha diversity analysis also showed no significant differences between fly species [Kruskal–Wallis test: Simpson index: *P *= 0.16; Shannon index: *P *= 0.11] (Fig. 2B and C), suggesting that the relative dominance of functions traits is similar across *Drosophila* host microbiomes.

**Fig. 3.**
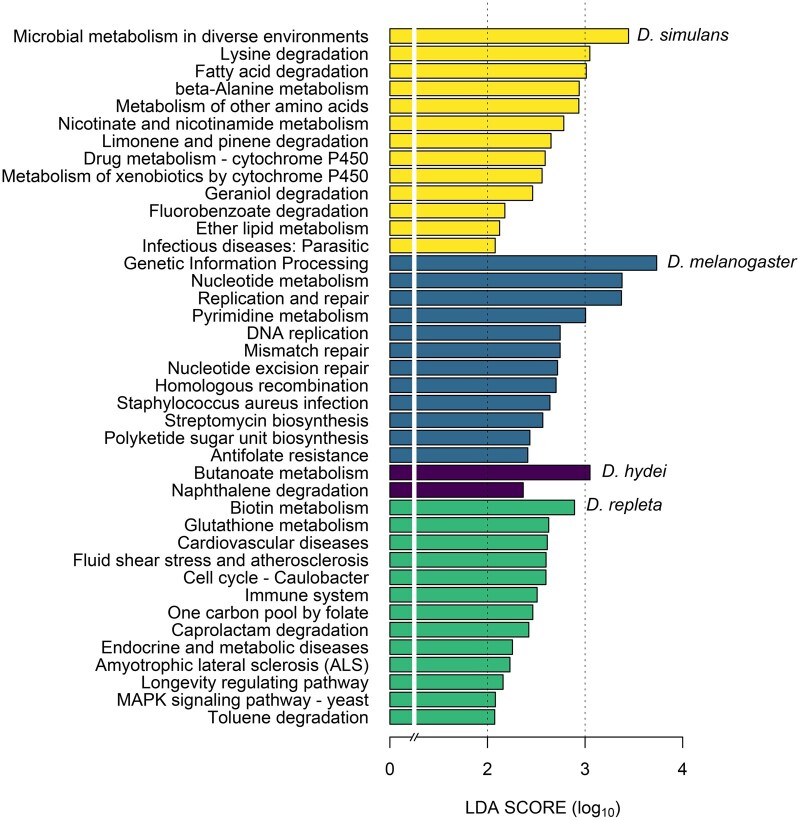
LEfSe analysis of the relative abundance of KEGG pathways at level 2 (with LDA scores greater than 2) between 4 wild *Drosophila* species.

We identified 332 KEGG functional pathways at level 3 associated with the reads obtained from 16S rRNA gene sequencing. Among these, the same 20 functions were consistently present in similar proportions across all species, collectively accounting for over 58% of the total relative abundance of functional traits in the microbiome of all *Drosophila* species (Table 1, Fig. 2D and Supplementary Fig. S3), indicating a high degree of functional redundancy among host microbiomes. LEfSe analysis with adjusted *P* values did not detect significant differences in the microbial functional traits among host species. However, when using unadjusted *P* values, we identified 58 functions with a strong predominance in different host species (LDA score > 2; Fig. 3). The gut microbiome of *D. simulans* was dominated by central energy-generating pathways, including microbial metabolism in diverse environments, lysine degradation, and fatty acid degradation. In contrast, the microbiome of *D. melanogaster* appeared to be more specialized in DNA replication and repair (eg genetic information processing, nucleotide metabolism, DNA replication), as well as antibiotics biosynthesis (streptomycin). The gut microbiome of *D. hydei*, was enriched in nutrient metabolism (eg butanoate), along with resistance mechanisms (naphthalene degradation). Finally, the gut microbiome in *D. repleta* was better characterized by its versatile range of pathways involved in nutrients biosynthesis and degradation (biotin, glutathione and folate), as well its capacity to sense and respond to the environment changes (MAPK signaling pathway − yeast).

**Table 1.** Relative abundance of the top 20 KEEG functional pathways (Level 3) in the gut microbiome of 4 wild *Drosophila* species

Level 3	Ds	Dm	Dh	Dr
**Metabolic pathways**	14.28 ± 0.25 ^a^	14.39 ± 0.22 ^a^	14.20 ± 0.22 ^a^	14.53 ± 0.31 ^a^
**Biosynthesis of secondary metabolites**	5.97 ± 0.15 ^a^	5.84 ± 0.25 ^a^	5.73 ± 0.11 ^a^	5.94 ± 0.11 ^a^
**ABC transporters**	5.54 ± 0.39 ^a^	6.10 ± 0.22 ^a^	5.51 ± 0.41 ^a^	5.71 ± 0.26 ^a^
**Microbial metabolism in diverse environments**	5.21 ± 0.16 ^a^	4.67 ± 0.13 ^a^	5.21 ± 0.12 ^a^	4.88 ± 0.07 ^a^
**Biosynthesis of antibiotics**	4.70 ± 023 ^a^	4.73 ± 0.18 ^a^	4.54 ± 0.12 ^a^	4.84 ± 0.13 ^a^
**Two-component system**	3.93 ± 0.42 ^a^	3.43 ± 0.26 ^a^	4.01 ± 0.13 ^a^	3.61 ± 0.30 ^a^
**Biosynthesis of amino acids**	2.64 ± 0.11 ^a^	2.98 ± 0.28 ^a^	2.62 ± 0.02 ^a^	2.63 ± 0.08 ^a^
**Quorum sensing**	2.47 ± 0.14 ^a^	2.44 ± 0.11 ^a^	2.40 ± 0.12 ^a^	2.72 ± 0.10 ^a^
**Carbon metabolism**	2.35 ± 0.05 ^a^	2.29 ± 0.01 ^a^	2.31 ± 0.02 ^a^	2.28 ± 0.02 ^a^
**Phosphotransferase system (PTS)**	1.34 ± 0.35 ^a^	3.01 ± 0.74 ^a^	2.18 ± 0.28 ^a^	1.30 ± 0.35 ^a^
**Purine metabolism**	1.50 ± 0.07 ^a^	1.76 ± 0.07 ^a^	1.59 ± 0.06 ^a^	1.66 ± 0.05 ^a^
**Amino sugar and nucleotide sugar metabolism**	1.20 ± 0.16 ^a^	1.86 ± 0.31 ^a^	1.48 ± 0.15 ^a^	1.19 ± 0.16 ^a^
**Glycolysis/Gluconeogenesis**	1.16 ± 0.07 ^a^	1.46 ± 0.21 ^a^	1.28 ± 0.08 ^a^	1.16 ± 0.08 ^a^
**Starch and sucrose metabolism**	0.83 ± 0.19 ^a^	1.91 ± 0.40 ^a^	1.08 ± 0.16 ^a^	0.97 ± 0.16 ^a^
**Pyruvate metabolism**	1.14 ± 0.06 ^a^	1.18 ± 0.02 ^a^	1.22 ± 0.06 ^a^	1.12 ± 0.04 ^a^
**Ribosome**	1.00 ± 0.07 ^a^	1.38 ± 0.09 ^a^	1.07 ± 0.07 ^a^	1.14 ± 0.06 ^a^
**Pyrimidine metabolism**	1.10 ± 0.04 ^ab^	1.26 ± 0.05 ^a^	1.06 ± 0.05 ^b^	1.10 ± 0.03 ^ab^
**Cysteine and methionine metabolism**	0.93 ± 0.08 ^a^	1.07 ± 0.10 ^a^	0.89 ± 0.03 ^a^	0.86 ± 0.04 ^a^
**Fructose and mannose metabolism**	0.75 ± 0.14 ^a^	1.10 ± 0.25 ^a^	0.98 ± 0.09 ^a^	0.70 ± 0.06 ^a^
**Glycine, serine and threonine metabolism**	0.82 ± 0.04 ^a^	0.86 ± 0.07 ^a^	0.73 ± 0.01 ^a^	0.80 ± 0.03 ^a^

Values are presented as the mean ± SE. Within each row, values sharing the same letter do not differ significantly at *P *< 0.05 as determined by Dunn’s test. The fly species are represented as follows: Ds (*D. simulans*), Dm (*D. melanogaster*), Dh (*D. hydei*), and Dr (*D. repleta*).

### Fitness in *D. simulans* and *D. hydei*

**Fig. 4.**
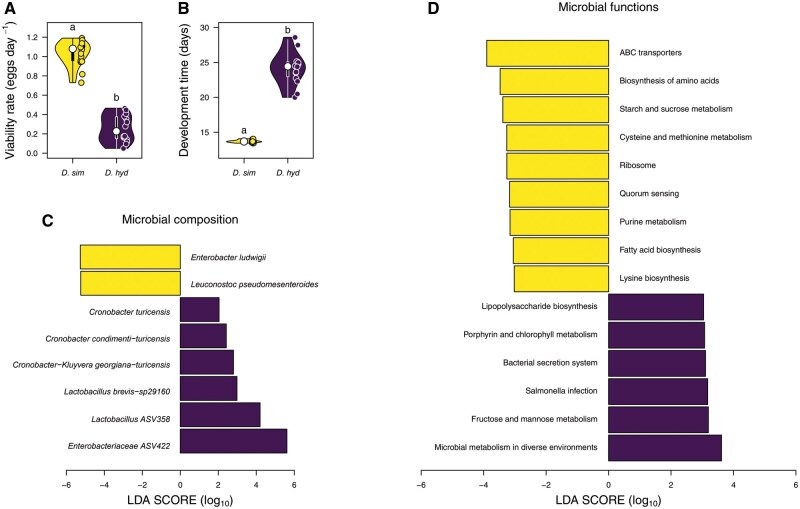
Comparison of fitness and microbial biomarkers at taxonomic and functional levels between *D. simulans* and *D. hydei*. A) Viability rate and B) development time were measured as indicators of fitness. C) LEfSe analysis showing the microbial taxonomic biomarkers between *D. simulans* and *D. hydei*. D) LEfSe analysis of microbial functions at level 3 (LDA > 3) between the 2 fly species.

*D. simulans* exhibited a clear fitness advantage over *D. hydei* (Fig. 4). Specifically, the average egg viability rate of *D. simulans* was significantly higher than that of *D. hydei* [Wilcoxon test: 𝑃 < 0.01], with a mean of 1.03 ± 0.03 eggs day^−1^ compared to 0.25 ± 0.04 eggs day^−1^. Additionally,*D. simulans* developed markedly faster than *D. hydei* [Wilcoxon test: 𝑃 < 0.01], reaching the adult stage in an average of 13.69-d, whereas *D. hydei* required around 24.45-d to complete development.

### Potential Microbiome Effects on Fitness of *D. simulans* and *D. hydei*

To identify bacterial biomarkers that might differentially affect fly fitness, we performed LEfSe analysis comparing the microbial communities of *D. simulans* and *D. hydei*. At the taxonomic level, *D. simulans* was characterized by 2 biomarkers: *Enterobacter ludwigii* and *Leuconostoc pseudomesenteroides*. In contrast, *D. hydei* exhibited 6 bacterial species with differential abundance, where *Enterobacteraceae ASV_422*, *Lactobacillus ASV_358* and *Lactobacillus brevis−sp29160* had the highest LDA scores (Fig. 4C). In terms of microbial functional profiles, *D. simulans* displayed nine functional biomarkers, with ABC transporters and biosynthesis of amino acids being the most abundant (Fig. 4D). Meanwhile, *D. hydei* showed 6 functional biomarkers, with microbial metabolism in diverse environments and fructose and mannose metabolism exhibiting the highest LDA scores (Fig. 4D).

A detailed analysis of microbial species and functions suggested that *D. simulans* harbored a greater number of bacteria and functional pathways that may enhance host fitness compared to *D. hydei* (Supplementary Table S2).

## Discussion

In this study, we advance the understanding of how the gut microbiome influences host performance by examining the microbial taxonomy and functionality across 4 *Drosophila* species sharing an ecological niche. By analyzing these variables, we sought to identify patterns that could reveal links between microbial community composition and host fitness outcomes. Our results provide a valuable framework for generating hypotheses regarding the broader ecological and evolutionary significance of the microbiome.

Overall, taxonomic analysis revealed differences in alpha and beta diversity between the *D. simulans*/*D. melanogaster* and *D. hydei*/*D. repleta* host groups, consistent with the concept of phylosymbiosis (Brooks et al. 2016). These findings differ from previous studies that did not detect phylosymbiotic signals in *Drosophila* species, which included 3 of the 4 species studied here (Adair et al. 2020, DuBose et al. 2025). The observed discrepancy could partly arise from differences in the methodological approach. Here, we studied host species with similar dietary niches, while prior studies focused on species with different diets (eg cactus vs. fruit feeders). Evolutionary divergence often promotes niche differentiation, especially in diet (Ackerly et al. 2006, Greene et al. 2020). In this way, the dietary diversification aligns with host phylogeny in some insect groups (eg cockroaches and termites), enhancing phylosymbiotic patterns (Dietrich et al. 2014). However, phylogeny and dietary niche do not have a clear relationship in *Drosophila* (Oliveira et al. 2012), which may affect the possibility of finding phylosymbiosis. This aligns with DuBose et al. (2025), who found that diet plays a more important role than host phylogeny in shaping microbial diversity. Yet diet al.ne does not fully explain discrepancies with Adair et al. (2020), who unexpectedly observed greater microbiome similarity between *D. melanogaster* and *D. hydei* than with *D. simulans*. These differences could result from laboratory rearing conditions, which are known to alter microbiome composition (Chandler et al. 2011, Brown et al. 2023). Notably, Adair et al. (2020) used antibiotics to eliminate intracellular bacteria such as *Wolbachia*. Thus, to further assess phylosymbiosis in *Drosophila* and resolve discrepancies between studies, we recommend studying wild populations with similar ecological niches to avoid confounding dietary and laboratory effects. It is important to note that we analyzed females of *D. hydei* and *D. repleta* and males for *D. simulans* and *D. melanogaster*, due to previous studies reported no sex-based differences in microbiome composition in *Drosophila* (Wong et al. 2013, Adair et al. 2020). Thus, results should be interpreted with caution, as subtle sex-related effects cannot be fully excluded here.

Our results also revealed that microbial functional diversity was similar among all fly species. Despite this broad functional redundancy, LEfSe analysis revealed a set of functional traits that underscore host-specific ecological interactions (Fig. 3). For instance, the gut microbiome of *D. simulans* is characterized by microbial metabolism in diverse environments and degradation of metabolites (eg lysine and fatty acid), suggesting a high metabolic plasticity that allows degrade different nutrients. Some of these functions may beneficiate the host by degrading toxic metabolites, such as geraniol and xenobiotics (Idda et al. 2020). In *D. melanogaster*, the dominance of genetic regulation pathways (eg genetic information processing, nucleotide metabolism, replication, and repair) and antibiotic biosynthesis (eg streptomycin) suggests an intense microbial competition within the gut ecosystem. The gut microbiome of *D. hydei* degrades toxic metabolites (eg naphthalene) and participate in the synthesis of nutrients (eg butanoate) that have been described as beneficial to the thermal tolerance of the host (Li et al. 2019), which indicate a positive effect on the host fitness. Finally, the microbiome of *D. repleta* was characterized by participate in the metabolism of nutrients (eg biotin, glutathione and folate), including their synthesis and degradation. Some of these nutrients are necessary for development (Storelli et al. 2011) and successful egg hatching (Douglas 2018), suggesting a role of microbiome on the host fitness. It is important to note that these functions were detected in LEfSe analysis only when using unadjusted *P*-values; after multiple-testing correction, no significant biomarkers remained (adjustment of *P*-values). Therefore, these trends should be considered exploratory and require confirmation in future studies.

In terms of fitness, *D. simulans* exhibited a higher egg-to-adult viability rate and faster development compared to *D. hydei*. The dominance of specific bacterial taxa may partly explain these differences. In this way, LEfSe analysis at the ASVs level revealed 2 bacterial biomarkers in *D. simulans* (*Enterobacter ludwigii* and *Leuconostoc pseudomesenteroides*; Fig. 4C), which have been reported to affect the fly’s fitness. For example, *D. melanogaster* monoassociated with *L. pseudomesenteroides* showed greater egg viability (∼40%) and development rate (∼11.5%) than germen-free flies (Whon et al. 2017, Pais et al. 2018). In contrast, *E. ludwigii* has been reported to reduce adults hatched in *D. melanogaster* by ∼10% without affecting developmental time (Priyadarsini et al. 2019), suggesting a relatively minor impact compared to *L. pseudomesenteroides*. On the other hand, *D. hydei* had 6 microbial biomarkers, where *Enterobacteraceae ASV_422*, *Lactobacillus ASV_358* and *Lactobacillus brevis−sp29160* showed elevated LDA scores (Fig. 4C). Previous studies reported that *Lactobacillus* genus decreases fecundity and developmental rate in *D. melanogaster* (Walters et al. 2020, Matthews et al. 2021), suggesting a similar effect on *D. hydei* fitness. In addition, the effect of *Cronobacter* genus has not been studied in *Drosophila*, however, some microorganisms of this genus are pathogens that decrease the offspring viability in some vertebrates (Hunter and Bean 2013, Fehr et al. 2015). Its presence suggests a possible detrimental effect on the host fitness and raises the hypothesis that *D. hydei* could act as a vector, as documented for other fly species (Pava-Ripoll et al. 2012). Interestingly, *D. hydei* harbored a higher relative abundance of *Acetobacter* (14.44%) compared to *D. simulans* (2.50%) (data not shown), a bacterial group that has been reported to improve development rate and fecundity on *D. melanogaster* (Walters et al. 2020, Matthews et al. 2021). This suggests that *Acetobacter* may not confer similar benefits in *D. hydei*, underscoring the importance of host–microbe specificity. Future studies are needed to clarify its functional role in this species.

The identified functional biomarkers may explain the differences in fitness observed between fly species. In this context, we identified 12 bacterial functions that could enhance *Drosophila* fitness, which 8 are present in *D. simulans* (Supplementary Table S2). For example, the biosynthesis of amino acids and fatty acids (Fig. 4D) have been reported to improve the fecundity (Henriques et al. 2020) and reduce development time (Shin et al. 2011) of *D. melanogaster*. Meanwhile, purine-metabolizing gut bacteria (eg *L. plantarum*) increase fly lifespan by preventing the accumulation of harmful purine-derived metabolites, such as uric acid (van Dam et al. 2020, Yamauchi et al. 2020). Additionally, lysine and arginine are essential amino acids known to increase fecundity (∼50%) (Grandison et al. 2009), development rate (Bayliak et al. 2018) and larvae-to-pupae viability (Consuegra et al. 2020) of *D. melanogaster*. By contrast, *D. hydei* had only 4 potentially beneficial functional biomarkers, with 2 directly related to fitness: folate and pantothenate metabolism (Supplementary Table S2). Specifically, larvae-to-pupae viability of *D. melanogaster* was high in diet with folate (∼85%) (Blatch et al. 2010) and pantothenate (∼86%) (Consuegra et al. 2020), compared to diets without folate (∼58%) or pantothenate (0%), respectively. Therefore, the smaller number of beneficial functions in *D. hydei* may partly explain its lower fitness relative to *D. simulans*. On the other hand, we identified 9 bacterial functions that may potentially decrease *Drosophila* fitness, 7 of which were present in *D. hydei* (Supplementary Table S3). These included degradation of beneficial compounds (eg porphyrin, lysine, and ascorbate) and pathogen-associated pathways (eg *Salmonella* infection, shigellosis) (Supplementary Table S3).

Taken together, our results reveal a strong correspondence between the taxonomic and functional characteristics of the gut microbiome with the phylogeny of the studied *Drosophila* species, consistent with the concept of phylosymbiosis. The phylogenetic niche conservatism hypothesis proposes that phylogenetically related host species should exhibit similar trophic niche (Losos 2008, Wiens et al. 2010), indicating comparable nutritional needs. Our findings suggest that, in the wild, closely related animal species filter microorganisms that perform similar functions to meet comparable nutritional requirements, highlighting the microbial association with the hosts’ evolutionary ecology. While our study examined a limited number of species, future research should incorporate a broader range of *Drosophila* species to determine whether these patterns hold across a wider phylogenetic spectrum. Additionally, we found that *D. simulans* exhibited higher fitness and greater abundance of bacterial species and functions that likely contribute to enhanced host reproduction and development. This observation aligns with phenological studies reporting higher abundance of *D. simulans* compared to *D. hydei* in central Chile (Benado and Brncic 1994, Alruiz et al. 2022) and the United States (Gleason et al. 2019) at similar latitudes (33° and 39°, respectively). It is important to note that diet may have contributed to the observed interspecific differences. For instance, wild-caught *D. hydei* and *D. simulans* differ in their carbon and nitrogen isotope ratios (Markow et al. 2000), indicating distinct dietary preferences and potentially different optimal diets for fitness. Because both species were reared under identical dietary conditions in our study, this setup may have favored *D. simulans* over *D. hydei*. Further studies are needed to evaluate this possibility.

Our study is the first to analyze the role of the gut microbiome in the competitive success between *Drosophila* species in the wild. Although we did not establish a direct causal link between the microbiome and host fitness, our results strongly suggest a microbial influence on the ecological interactions of hosts. To test these patterns, future studies should manipulate microbial composition or metabolites. This could have implications in applied areas such as insect pest control programs, opening the opportunity to investigate strategies targeting the microorganisms that influence fly fitness. Finally, *Drosophila* species, including *D. simulans* and *D. hydei*, exhibit changes in abundance across distribution gradients (Brncic 1992, Barker et al. 2005); therefore, further research should explore how life history traits vary between animals’ species across localities and whether the microbiome is associated with these traits.

## Supplementary Material

ieaf114_Supplementary_Data
