# Supplementary material for: Phylosymbiosis and functional redundancy in the Drosophila (Diptera: Drosophilidae) gut microbiome and its implications for host fitness
Source: J Insect Sci. 2026 Jan 27;26(1):ieaf114. doi: 10.1093/jisesa/ieaf114 (PMC12848231; doi:10.1093/jisesa/ieaf114)
Supplement: ieaf114_Supplementary_Data [file ieaf114_supplementary_data.docx]

Supplementary material for:

**Phylosymbiosis and functional redundancy in the *Drosophila* (Diptera: Drosophilidae) gut microbiome and its implications in host fitness**

**Supp Table S1.** Sequencing statistics for four *Drosophila* species. The statistics are indicated in each sample of each *Drosophila* species.

| Sample | Number of sequences | Good's coverage |
| --- | --- | --- |
| DsW1 | 11256 | 100 |
| DsW2 | 1079 | 100 |
| DsW3 | 245 | 100 |
| DsW4 | 83 | 100 |
| DsW5 | 18952 | 100 |
| DsF5.1 | 161 | 100 |
| DsF5.2 | 108 | 100 |
| DsF5.3 | 196 | 98.98 |
| DsF5.4 | 1589 | 100 |
| DsF5.5 | 1317 | 100 |
| DmW1 | 1015 | 99.90 |
| DmW2 | 477 | 99.79 |
| DmW3 | 1278 | 100 |
| DmW4 | 937 | 100 |
| DmW5 | 2106 | 99.95 |
| DhW1 | 4383 | 99.98 |
| DhW2 | 12592 | 99.98 |
| DhW3 | 847 | 99.88 |
| DhW4 | 930571 | 100 |
| DhW5 | 5521 | 99.95 |
| DrW1 | 8722 | 100 |
| DrW2 | 202083 | 100 |
| DrW3 | 1346 | 100 |
| DrW4 | 14706 | 100 |
| DrW5 | 527300 | 100 |
| DhF5.1 | 660325 | 100 |
| DhF5.2 | 13379980 | 100 |
| DhF5.3 | 3977695 | 100 |
| DhF5.4 | 5963266 | 100 |
| DhF5.5 | 11963269 | 100 |

DsW (*D. simulans* wild), DsF5 (*D. simulans* F5), DmW (*D. melanogaster* wild), DhW (*D. hydei* wild), DrW (*D. repleta* wild) and DhF5 (*D. hydei* F5).

**Supp Table S2**. Microbial functions detected by LEfSe analysis that have been reported to improve the host fitness.

| Function | LDA score | Host species that contains the microbial function | Effects of microbial function on fly fitness | Reference |
| --- | --- | --- | --- | --- |
| Biosynthesis of amino acids | 3.47 | *D. simulans* | Increase fecundity. | (Henriques et al. 2020) |
| Purine metabolism | 3.15 | *D. simulans* | Increase lifespan preventing the accumulation of purine-derived metabolites. | (van Dam et al. 2020, Yamauchi et al. 2020) |
| Fatty acid biosynthesis | 3.05 | *D. simulans* | Reduces development time. | (Shin et al. 2011) |
| Lysine biosynthesis | 3.02 | *D. simulans* | Increase larvae-to-pupae viability and fecundity. | (Grandison et al. 2009, Consuegra et al. 2020) |
| Arginine biosynthesis | 2.70 | *D. simulans* | Increase larvae-to-pupae viability, fecundity and development rate. | (Grandison et al. 2009, Bayliak et al. 2018, Consuegra et al. 2020) |
| Dioxin degradation | 2.40 | *D. simulans* | Prevents the toxic effects of dioxins. | (Çolak and Uysal 2021) |
| Thyroid hormone synthesis | 2.31 | *D. simulans* | Insect juvenile hormone (JH) shares structural and functional similarities to mammalian thyroid hormones. The microbiome increases eggs production by regulating JH. | (Flatt et al. 2006, Chu et al. 2025) |
| Atrazine degradation | 2.27 | *D. simulans* | Atrazine is an herbicide. The gut microbiome enhances the host’s tolerance to atrazine. | (Brown et al. 2021) |
| Folate biosynthesis | 2.67 | *D. hydei* | Improve larvae-to-pupae viability. | (Blatch et al. 2010) |
| Metabolism of xenobiotics by cytochrome P450 | 2.39 | *D. hydei* | Prevents the toxic effects of xenobiotics. | (Idda et al. 2020) |
| Pantothenate and CoA biosynthesis | 2.38 | *D. hydei* | Increase larvae-to-pupae viability and fecundity. | (Consuegra et al. 2020, Serrato-Salas and Gendrin 2023) |
| Geraniol degradation | 2.25 | *D. hydei* | Geraniol is a plants’ defensive chemical, and the microbiome help detoxify it. | (Francoeur et al. 2020) |

**Supp Table S3.** Microbial functions detected by LEfSe analysis that have been reported to impair the host fitness.

| Function | LDA score | Host species that contains the microbial function | Effects of microbial function on fly fitness | Reference |
| --- | --- | --- | --- | --- |
| Prodigiosin biosynthesis | 2.63 | *D. simulans* | Prodigiosin is a microbial metabolite with insecticidal effects. | (Liang et al. 2013) |
| Staphylococcus aureus infection | 2.43 | *D. simulans* | *Staphylococcus aureus* deceases fly survival. | (Needham et al. 2004) |
| Salmonella infection | 3.17 | *D. hydei* | *Salmonella* deceases fly survival. | (Brandt et al. 2004) |
| Lysine degradation | 2.90 | *D. hydei* | Lysine increases larvae-to-pupae viability and fecundity. Its degradation decreases their availability to the host. | (Grandison et al. 2009, Consuegra et al. 2020) |
| Ascorbate and aldarate metabolism | 2.90 | *D. hydei* | Ascorbate increases fly lifespan. Microbial metabolism of ascorbate may reduce its availability to the host. | (Suh et al. 2017) |
| Nicotinate and nicotinamide metabolism | 2.77 | *D. hydei* | Nicotinate improves larvae-to-pupae viability. Its degradation decreases its availability to the host. | (Consuegra et al. 2020) |
| Glutathione metabolism | 2.69 | *D. hydei* | Glutathione is an antioxidant that decrease the survival of pathogens. Its degradation promotes the proliferation of pathogens. | (Brouwer et al. 2022) |
| Shigellosis | 2.43 | *D. hydei* | *Shigella* infected flies shows metabolic disorders. | (Najjar et al. 2022) |
| Aminobenzoate degradation | 2.28 | *D. hydei* | Para-aminobenzoic acid (PABA) is used to synthesize folic acid. Its degradation reduces its availability, which may negatively affect the host. | (Smith et al. 2015) |

**Supp Fig. S1.** Rarefaction curves of the bacterial communities in four *Drosophila* species. The rarefied curves indicate that the majority of amplicon sequence variants (ASVs) were detected. DsWild (*D. simulans* wild), DsF5 (*D. simulans* F5), DmWild (*D. melanogaster* wild), DhWild (*D. hydei* wild), DrWild (*D. repleta* wild) and DhF5 (*D. hydei* F5).
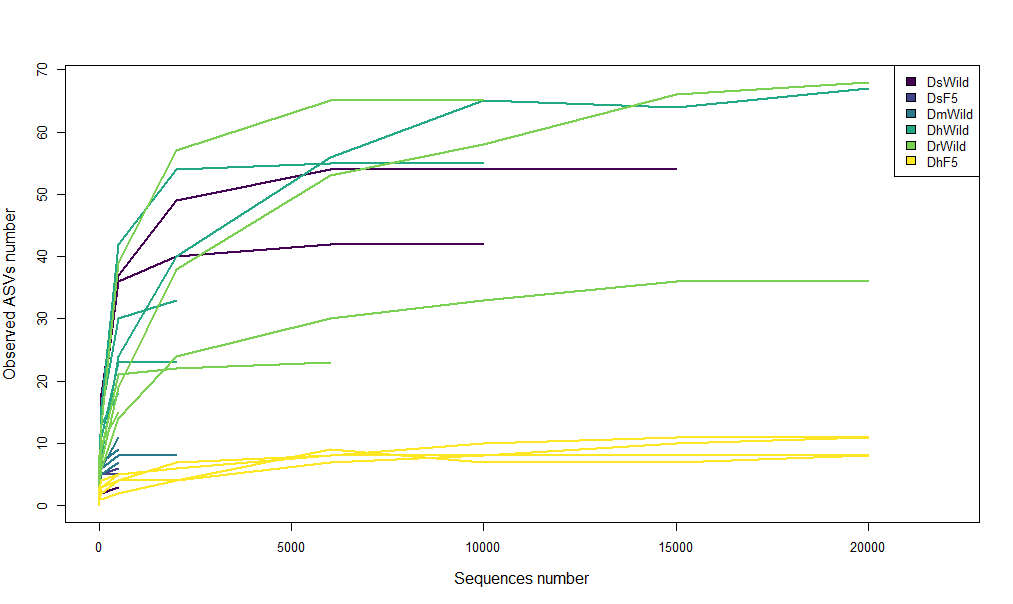


**Supp Fig. S2.** Relative abundance of the 30 most abundant bacterial genera across individual samples of each *Drosophila* species. Five biological replicates (S1-S5) were analyzed per species.
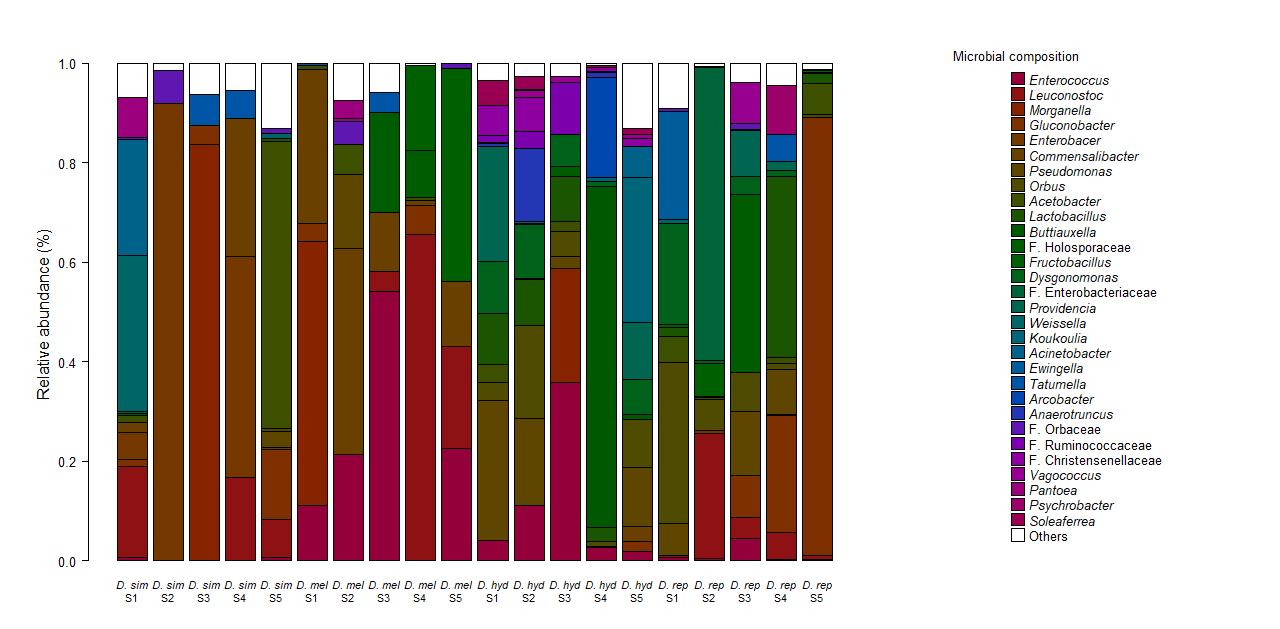


**Supp Fig. S3.** Relative abundance of the 30 most abundant predicted bacterial functions across individual samples of each *Drosophila* species. Five biological replicates (S1-S5) were analyzed per species.


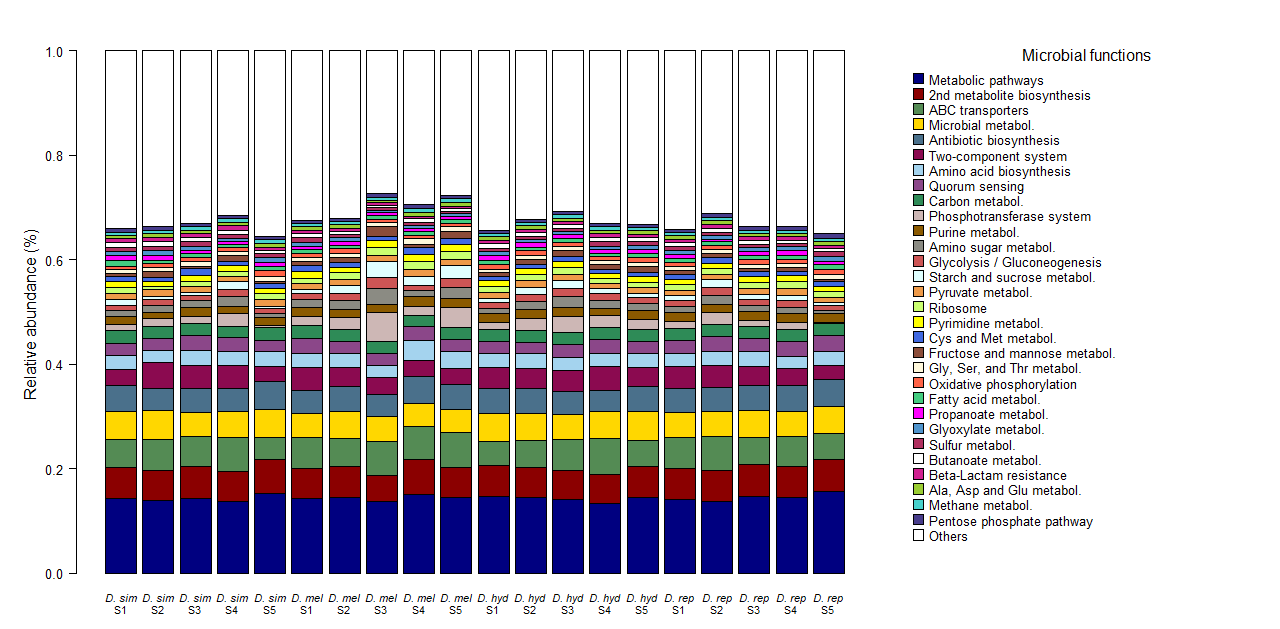


**References Cited in Supplementary Material**

Bayliak MM, Lylyk MP, Maniukh O V., et al. 2018. Dietary l-arginine accelerates pupation and promotes high protein levels but induces oxidative stress and reduces fecundity and life span in Drosophila melanogaster. J Comp Physiol B. 188(1). https://doi.org/10.1007/s00360-017-1113-6

Blatch SA, Meyer KW, Harrison JF. 2010. Effects of dietary folic acid level and symbiotic folate production on fitness and development in the fruit fly drosophila melanogaster. Fly (Austin). 4(4). https://doi.org/10.4161/fly.4.4.13258

Brandt SM, Dionne MS, Khush RS, et al. 2004. Secreted bacterial effectors and host-produced eiger/TNF drive death in a Salmonella-infected fruit fly. PLoS Biol. 2(12). https://doi.org/10.1371/journal.pbio.0020418

Brouwer S, Jespersen MG, Ong CLY, et al. 2022. Streptococcus pyogenes Hijacks Host Glutathione for Growth and Innate Immune Evasion. mBio. 13(3). https://doi.org/10.1128/mbio.00676-22

Brown JB, Langley SA, Snijders AM, et al. 2021. An integrated host-microbiome response to atrazine exposure mediates toxicity in Drosophila. Commun Biol. 4(1). https://doi.org/10.1038/s42003-021-02847-y

Chu B, Ge S, He W, et al. 2025. Gut symbiotic bacteria enhance reproduction in Spodoptera frugiperda (J.E. Smith) by regulating juvenile hormone III and 20-hydroxyecdysone pathways. Microbiome. 13(1). https://doi.org/10.1186/s40168-025-02121-x

Çolak DA, Uysal H. 2021. Determination of the genotoxic effects of various dioxins by <em>Drosophila </em>wing spot test. Indian J Exp Biol. https://doi.org/10.56042/ijeb.v59i09.54951

Consuegra J, Grenier T, Baa-Puyoulet P, et al. 2020. Drosophila-associated bacteria differentially shape the nutritional requirements of their host during juvenile growth. PLoS Biol. 18(3). https://doi.org/10.1371/journal.pbio.3000681

van Dam E, van Leeuwen LAG, dos Santos E, et al. 2020. Sugar-induced obesity and insulin resistance are uncoupled from shortened survival in Drosophila. Cell Metab. 31(4). https://doi.org/10.1016/j.cmet.2020.02.016

Flatt T, Moroz LL, Tatar M, et al. 2006. Comparing thyroid and insect hormone signaling. In: Integrative and Comparative Biology. Vol. 46. https://doi.org/10.1093/icb/icl034

Francoeur CB, Khadempour L, Moreira-Soto RD, et al. 2020. Bacteria contribute to plant secondary compound degradation in a generalist herbivore system. mBio. 11(5). https://doi.org/10.1128/mBio.02146-20

Grandison RC, Piper MDW, Partridge L. 2009. Amino-acid imbalance explains extension of lifespan by dietary restriction in Drosophila. Nature. 462(7276). https://doi.org/10.1038/nature08619

Henriques SF, Dhakan DB, Serra L, et al. 2020. Metabolic cross-feeding in imbalanced diets allows gut microbes to improve reproduction and alter host behaviour. Nat Commun. 11(1). https://doi.org/10.1038/s41467-020-18049-9

Idda T, Bonas C, Hoffmann J, et al. 2020. Metabolic activation and toxicological evaluation of polychlorinated biphenyls in Drosophila melanogaster. Sci Rep. 10(1). https://doi.org/10.1038/s41598-020-78405-z

Liang TW, Chen SY, Chen YC, et al. 2013. Enhancement of prodigiosin production by serratia marcescens TKU011 and its insecticidal activity relative to food colorants. J Food Sci. 78(11). https://doi.org/10.1111/1750-3841.12272

Najjar H, Al-Ashmar S, Qush A, et al. 2022. Enteric pathogens modulate metabolic homeostasis in the Drosophila melanogaster host. Microbes Infect. 24(4). https://doi.org/10.1016/j.micinf.2022.104946

Needham AJ, Kibart M, Crossley H, et al. 2004. Drosophila melanogaster as a model host for Staphylococcus aureus infection. Microbiology (N Y). 150(7). https://doi.org/10.1099/mic.0.27116-0

Serrato-Salas J, Gendrin M. 2023. Involvement of Microbiota in Insect Physiology: Focus on B Vitamins. mBio. 14(1). https://doi.org/10.1128/mbio.02225-22

Shin SC, Kim SH, You H, et al. 2011. Drosophila microbiome modulates host developmental and metabolic homeostasis via insulin signaling. Science (1979). 334(6056). https://doi.org/10.1126/science.1212782

Smith TA, Driscoll T, Gillespie JJ, et al. 2015. A Coxiella-like endosymbiontis a potential vitamin source for the lone star tick. Genome Biol Evol. 7(3). https://doi.org/10.1093/gbe/evv016

Suh HJ, Shin B, Han SH, et al. 2017. Behavioral changes and survival in Drosophila melanogaster: Effects of ascorbic acid, taurine, and caffeine. Biol Pharm Bull. 40(11). https://doi.org/10.1248/bpb.b17-00321

Yamauchi T, Oi A, Kosakamoto H, et al. 2020. Gut bacterial species distinctively impact host purine metabolites during aging in Drosophila. iScience. 23(9). https://doi.org/10.1016/j.isci.2020.101477
